# 3DGAUnet: 3D Generative Adversarial Networks with a 3D U-Net Based Generator to Achieve the Accurate and Effective Synthesis of Clinical Tumor Image Data for Pancreatic Cancer

**DOI:** 10.3390/cancers15235496

**Published:** 2023-11-21

**Authors:** Yu Shi, Hannah Tang, Michael J. Baine, Michael A. Hollingsworth, Huijing Du, Dandan Zheng, Chi Zhang, Hongfeng Yu

**Affiliations:** 1 School of Computing, University of Nebraska-Lincoln, Lincoln, NE 68588, USA; yu.shi@huskers.unl.edu (Y.S.); htang11@unl.edu (H.T.); 2 School of Biological Sciences, University of Nebraska-Lincoln, Lincoln, NE 68588, USA; 3 Complex Biosystems Program, University of Nebraska-Lincoln, Lincoln, NE 68588, USA; 4 Department of Radiation Oncology, University of Nebraska Medical Center, Omaha, NE 68198, USA; mbaine@unmc.edu; 5 Eppley Institute for Research in Cancer and Allied Diseases, University of Nebraska Medical Center, Omaha, NE 68198, USA; mahollin@unmc.edu; 6 Department of Mathematics, University of Nebraska-Lincoln, Lincoln, NE 68588, USA; hdu@unl.edu; 7 Department of Radiation Oncology, University of Rochester Medical Center, Rochester, NY 14626, USA; dandan_zheng@urmc.rochester.edu; 8 Holland Computing Center, University of Nebraska-Lincoln, Lincoln, NE 68588, USA

**Keywords:** pancreatic cancer, generative adversarial network, 3D volume synthesis, clinical tumor, medical imaging

## Abstract

**Simple Summary:**

Pancreatic ductal adenocarcinoma (PDAC) has the most elevated fatality rate among the primary types of solid malignancies, posing an urgent need for early detection of PDAC to improve survival rates. Recent progress in medical imaging and computational algorithms provides potential solutions, with deep learning, particularly convolutional neural networks (CNNs), showing promise. However, progress is hindered by a lack of clinical data. This study introduces a new model, 3DGAUnet, employing generative adversarial networks (GANs) to generate realistic 3D CT images of PDAC. In contrast to conventional 2D models, 3DGAUnet maintains contextual information between slices, leading to substantial improvements in efficiency and accuracy. The key innovation lies in integrating a 3D U-Net architecture into the generator, augmenting the learning of shape and texture for PDAC tumors and pancreatic tissue. Thorough validation demonstrates the model’s efficacy across diverse datasets, presenting a promising solution to overcome data scarcity, enhance synthesized data quality, and advance deep learning for accurate PDAC detection, with broader implications for other solid tumors in medical imaging.

**Abstract:**

Pancreatic ductal adenocarcinoma (PDAC) presents a critical global health challenge, and early detection is crucial for improving the 5-year survival rate. Recent medical imaging and computational algorithm advances offer potential solutions for early diagnosis. Deep learning, particularly in the form of convolutional neural networks (CNNs), has demonstrated success in medical image analysis tasks, including classification and segmentation. However, the limited availability of clinical data for training purposes continues to represent a significant obstacle. Data augmentation, generative adversarial networks (GANs), and cross-validation are potential techniques to address this limitation and improve model performance, but effective solutions are still rare for 3D PDAC, where the contrast is especially poor, owing to the high heterogeneity in both tumor and background tissues. In this study, we developed a new GAN-based model, named 3DGAUnet, for generating realistic 3D CT images of PDAC tumors and pancreatic tissue, which can generate the inter-slice connection data that the existing 2D CT image synthesis models lack. The transition to 3D models allowed the preservation of contextual information from adjacent slices, improving efficiency and accuracy, especially for the poor-contrast challenging case of PDAC. PDAC’s challenging characteristics, such as an iso-attenuating or hypodense appearance and lack of well-defined margins, make tumor shape and texture learning challenging. To overcome these challenges and improve the performance of 3D GAN models, our innovation was to develop a 3D U-Net architecture for the generator, to improve shape and texture learning for PDAC tumors and pancreatic tissue. Thorough examination and validation across many datasets were conducted on the developed 3D GAN model, to ascertain the efficacy and applicability of the model in clinical contexts. Our approach offers a promising path for tackling the urgent requirement for creative and synergistic methods to combat PDAC. The development of this GAN-based model has the potential to alleviate data scarcity issues, elevate the quality of synthesized data, and thereby facilitate the progression of deep learning models, to enhance the accuracy and early detection of PDAC tumors, which could profoundly impact patient outcomes. Furthermore, the model has the potential to be adapted to other types of solid tumors, hence making significant contributions to the field of medical imaging in terms of image processing models.

## 1. Introduction

Pancreatic ductal adenocarcinoma (PDAC) represents a significant public health concern, due to its delayed identification, the restricted efficacy of current chemotherapeutic treatments, and poor overall prognosis. It has the most elevated fatality rate among the primary types of solid malignancies. Despite extensive clinical and research endeavors spanning decades, the one-year survival rate stands at 20%, while the five-year survival rate remained in the single digits for a considerable time and only recently improved to 11% [1]. Despite the potential for a substantial increase in the 5-year relative survival rate to 42% [2] if early detection at the localized stage is achieved, there is currently a lack of definitive screening methods for reliably identifying early-stage pancreatic cancer in asymptomatic individuals.

Computed tomography (CT) is one of the primary diagnostic imaging methods. In recent years, deep-learning-based methods have increasingly been perceived as versatile applications. They can directly integrate physical and semantic details into neural network architectures [3,4,5,6,7] and are employed to solve computer vision tasks in medical imaging, such as segmentation, registration, and classification of chest X-rays and tissue histopathology images [8,9]. For example, convolutional neural network (CNN) models have shown high feasibility in image classification tasks, in both natural and medical images, from 2D models to 3D models [10,11,12]. Some similar studies have been applied for pancreatic cancer classifiers to analyze and interpret features from medical imaging data [13,14].

During the development of a deep learning model for image tasks, a substantial dataset (e.g., thousands of images) is typically needed, to ensure the model converges without overfitting. Nevertheless, the availability of clinical information, particularly for PDAC, is frequently constrained by the small size of the cohorts, which presents obstacles to achieving optimal model training. Researchers have developed methods such as data augmentation, generative adversarial networks (GAN), cross-validation, and optimization approaches like sharp-aware minimization [15] to overcome the lack of training data. Generative models have demonstrated efficacy in medical image synthesis, particularly in 2D imaging modalities. Recently, researchers have developed 2D-based GAN models to generate realistic CT images of pancreatic tumors [16,17]. Nevertheless, the utilization of 3D generative models in the context of PDAC is still constrained, and directly applying existing approaches (e.g., 3D-GAN [18]) may not lead to desirable results for synthesizing three-dimensional CT image data specific to PDAC. PDAC tumors often exhibit subtle imaging features, because they can be iso-attenuating or hypodense compared to the surrounding pancreatic tissue, making them difficult to distinguish visually. Additionally, PDAC tumors may lack well-defined margins, making differentiating them from normal pancreatic parenchyma challenging. Therefore, developing efficient techniques for enhancing 3D PDAC tumor datasets is crucial, to facilitate the progress of deep learning models in addressing PDAC.

In this work, we develop a GAN-based tool capable of generating realistic 3D CT images depicting PDAC tumors and pancreas tissue. To overcome these challenges and make this 3D GAN model perform better, our innovation was to develop a 3D U-Net architecture for the generator, to improve shape and texture learning for PDAC tumors and pancreatic tissue. The application of 3D U-Net in medical picture auto-segmentation showed appropriate and superior results. Notably, this is the first instance of its integration into GAN models. This 3D GAN model generates volumetric data of PDAC tumor tissue CT images and healthy pancreas tissue CT images separately, and a blending method was employed to create realistic final images. Thorough examination and validation across many datasets were conducted on the developed 3D GAN model, to ascertain the efficacy and applicability of the model in clinical contexts. We evaluated the effectiveness of our approach by training a 3D CNN model with synthetic image data, to predict 3D tumor patches. A software package, 3DGAUnet, was developed to implement this 3D generative adversarial network with a 3D U-Net-based generator for tumor CT image synthesis. This package has the potential to be adapted to other types of solid tumors, hence making significant contributions to the field of medical imaging in terms of image processing models. This software package is available at https://github.com/yshi20/3DGAUnet (accessed on 12 October 2023).

## 2. Materials and Methods

**Figure 1:**
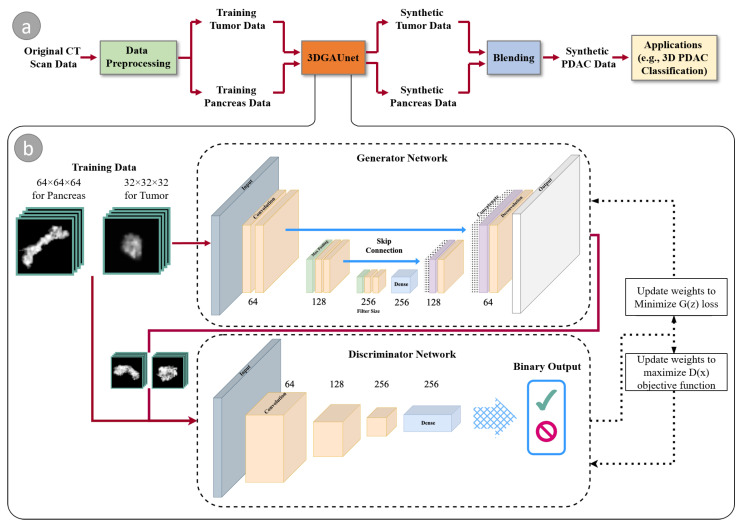
An overview of our method. (**a**) the workflow components and (**b**) the architecture of our GAN-based model, 3DGAUnet, consisting of a 3D U-Net-based generator network and a 3D CNN-based discriminator network to generate synthetic data.

Figure 1a illustrates the overall workflow of our proposed method. Given a set of PDAC CT images that can be acquired through different sources, we first conduct data preprocessing on these raw image data to tackle data heterogeneity and generate normalized and resampled volume data for tumor tissues and pancreas. These preprocessed datasets are then used as the training set and fed into 3DGAUnet, the new 3D GAN model developed in this work for tumor CT image synthesis. After the tumor and pancreas types are learned independently via 3DGAUnet, the corresponding synthetic data can be generated.

To effectively combine these synthetic tissues, we evaluated three blending methods and identified the most suitable technique for PDAC tumor CT images. Given that the pancreas is a parenchymal organ, the relative location of the tumor tissue was found to be less significant. As a result, the focus was primarily on blending the different tissue types seamlessly and realistically to ensure accurate and reliable results for diagnosing PDAC tumors in CT images.

We evaluated the usability of the synthetic data by applying it in a diagnosis task. For this purpose, we employed a 3D CNN classifier capable of taking 3D volumes as input, which was an improvement over the traditional classification tools that only use individual slices and overlook the inter-slice information.

By integrating the synthetic data, we addressed common challenges encountered in real-world scenarios, such as the small size of the dataset and imbalanced data. The addition of synthetic samples helped to improve the model’s performance and mitigate issues related to imbalanced datasets.

### 2.1. 3D CT Image Data Preprocessing

We used a training dataset for the GAN model, consisting of PDAC CT images from 174 patients from two sources. One was from the University of Nebraska Medical Center (UNMC) rapid autopsy program (RAP). This dataset has 71 de-identified patient data points, with the tumor contour labeled by clinical professionals (UNMC IRB PROTOCOL #127-18-EP). The other was the Medical Segmentation Decathlon pancreas training data [19], which has 103 volumetric images with a segmentation mask of tumors and pancreas. In addition, the training dataset consisted of 80 healthy pancreas CT images from the Cancer Imaging Archive (TCIA) Pancreas-CT data [20].

Normalization and resampling were essential for these raw image data, as the images were obtained from various instruments with distinct configurations. By resampling all volumetric data to 1 mm isotropic voxel spacing, each pixel in an image represents the same physical distance along each axis. For normalization, the window level threshold is an important measurement. In CT imagery, the Hounsfield unit (HU) is used as a dimensionless unit to measure radio density and quantify tissues within the body. It is calculated based on a linear transformation of X-ray’s baseline linear attenuation coefficient, where distilled water is defined as zero HU and air is defined as −1000 HU [21]. Similar HU values across different studies indicate the same type of tissue. However, calculating HU values for grayscale images with different window-level settings can lead to different visual appearances. In this study, the original images had HU values ranging from −408 to 1298. For normalization, we mapped HU values to the range of −100 to 170 for abdominal soft tissues based on the advice of an experienced radiologist.

The original image data from UNMC had metal markers that caused extremely high HU values and deflected the X-ray beam, causing the sounding tissue to have a higher HU value. To counter this defect, pixels above 200 HU were replaced with the mean HU value of the entire pancreas captured in each CT scan. Since the pancreas has an irregular shape, the tumor region and surrounding pancreas tissues that filled a cube with 64×64×64 pixels were kept as the field of interest. This made the GAN model learn the texture instead of learning the premier of the pancreas organ, which varies among different patients. After the data preprocessing step, the training volumetric data were 32×32×32 for tumor tissue and 64×64×64 pancreas cubes in grayscale.

### 2.2. 3DGAUnet: 3D U-Net Based GAN Model

We devised a 3D U-Net-based GAN model, named 3DGAUnet, to synthesize 3D pancreas tumor and tissue images. Figure 1b shows our model architecture. At a high level, our model follows a typical GAN model that comprises two primary components, a generator *G*, and a discriminator *D*. The generator creates synthetic samples, while the discriminator differentiates between synthetic and natural samples. They compete in an adversarial game to improve the generator’s ability to generate genuine samples and the discriminator’s ability to identify them. The aim is to produce synthetic samples that closely approximate their natural counterparts. This process can be defined as a min-max optimization task

(1) $$min_{G}max_{D}L_{GAN},$$

and $L_{GAN}$ is defined as

(2) $$L_{GAN}=E_{x∼p_{data}}\left[\log D\left(x\right)\right]+E_{z∼p_{z}}\left[\log\left(1-D\left(G\left(z\right)\right)\right)\right]$$

where E is the cross-entropy of the binary classifier of the discriminator. The task of the generator *G* is to minimize the generator loss to generate synthesized images that cannot be distinguished by the discriminator *D*:

(3) $$min_{G}L_{GAN}=min_{G}E_{Z∼p_{Z}}\left[\log\left(1-D\left(G\left(z\right)\right)\right)\right]$$

The task of the discriminator *D* is to better separate real images and synthesized images:

(4) $$max_{D}L_{GAN}=max_{D}\;\left\{E_{x∼p_{data}}\left[\log D\left(x\right)\right]+E_{z∼p_{z}}\left[\log\left(1-D\left(G\left(z\right)\right)\right)\right]\right\}.$$

In our 3D GAN model, we employ a 3D U-Net-based structure for the 3D GAN generator and a 3D CNN-based classifier as the discriminator. The 3D U-Net structure has proven advantages for effectively capturing both global and local structures, such as for CT image auto-segmentation for tissues like the pancreas [22]. We developed a 3D U-Net structure for 3D image synthesis, and to the best of our knowledge, this is the first time that a 3D U-Net structure has been used for a generator in a GAN model to tackle the challenging shape and texture learning for PDAC tumors and pancreatic tissue. Each convolution layer in this model has a kernel size of 3×3×3, a stride of 2, and a ReLU activation. The skip connections allow the low-level information to be passed to the upsampling stacks, to avoid the vanishing gradient problem.

The discriminator is used to identify if the input image is synthesized images from the generator’s output. It has three 3D convolutional blocks, and each block starts with a 3D convolution with a kernel size of 2×2×2 and sides of 1, followed by a 3D max-pooling layer with a pool size of 2, and batch normalization. After three 3D convolutional blocks, this is flattened to a fully connected dense layer, and binary output is generated by the sigmoid function.

Our approach can be used to separately train 3D models of tumors and pancreatic healthy tissue. The training procedure was optimized with respect to discriminator loss. A total of 500 3D tumor and pancreas volumes were synthesized. By inserting tumor tissue into the pancreas cube, a 3D volume of the tumor with surrounding pancreas tissues could be generated.

### 2.3. Blending to Create PDAC Tissues

Because the tumor and pancreas tissue volumes were generated separately, it was essential to consider how to combine the tumor and pancreas volumes to create realistic tumorous pancreas tissue. We experimented with and compared three blending methods for merging the tumor and tissue volumes of a pancreas. The first method (namely Blend I) was a straightforward copy-and-paste operation that used the tumor voxels to replace the corresponding pancreas tissue voxels. Our second and third methods (namely Blend II and Blend III, respectively) were inspired by DeepImageBlending, a deep learning technique that improves Poisson image blending [23]. Deep image blending is a two-stage image blending algorithm. First, it generates a seamless boundary for the source region, to eliminate visible seams. Then, it refines the source region by matching styles and textures with the target image. The algorithm uses a differentiable loss function based on the Poisson equation and can handle various image styles, including stylized paintings. It achieves visually consistent blending without relying on training data. Our Blend II and Blend III methods are the first and second stages of the tool, respectively. The motivation for comparing the two stages was because, in a natural image blending task, the object should look natural and share a similar style with the background image, but this might not be true for a CT image. Unlike natural images that are acquired from light reflection from objects, CT images are created by recording the X-ray beam attenuation from different directions, and therefore a presumption of a similar style may not be valid. To find the best blending method, we compared the three blending methods using visual inspection and Fréchet inception distance (FID) values. After synthetic tumors and healthy pancreas tissues were directly output by our 3D GAN model, they were blended to generate synthetic PDAC tissues with the best blending method. The comparison of the blending methods will be provided in Section 3.2.

### 2.4. Evaluation of Synthesized Images

The performance of our developed 3DGAUnet model was evaluated both qualitatively and quantitatively. We visualized the generated volumes with 2D cross-slices and 3D volume rendering for qualitative evaluation [24]. For quantitative evaluation, we used FID values on 2D slices [25].

We propose a 3D evaluation metric, called Fréchet 3D distance (F3D), for comparing the Fréchet distance of the activation layer from a 3D CNN network with the quality of the 3D GAN model.The distance *d* is calculated as

(5) $$d^{2}=\left|\right|\mu_{1}-\mu_{2}{\left|\right|}^{2}+Tr\left(C_{1}+C_{2}-2\sqrt{C_{1}C_{2}}\right),$$

where $\mu_{1}$ and $\mu_{2}$ are the feature-wise means of the real and synthesized images, $C_{1}$ and $C_{2}$ are the covariance matrix of the feature vectors for the real and synthesized images, and Tr is the trace linear algebra operation that is the sum of the elements along the main diagonal of the square matrix.

To calculate the μ and *C*, we needed the feature vector from the last pooling layer out of a pretrained neural network. The original FID used a trained Inception V3 model [26]. Our approach, instead, used a 3D CNN with 17 layers, including four 3D convolutional blocks with a fixed random state 42, and the feature vector was the flattened layer after the last convolutional block, having a length of 512. Samples were compared in batches, and then the μ and *C* could be calculated with the matrix that consists of the feature vector from each sample.

In addition, the quality of the images generated by our developed 3DGAUnet model was evaluated using the squared maximum mean discrepancy (MMD^2^), which employs kernel functions in the reproducing kernel Hilbert space to quantify the discrepancy between two distributions [27]. In this study, we also used pair-wise multi-scale structural similarity (MS-SSIM) to assess the diversity of the images generated by our 3DGAUnet model. MS-SSIM is a metric that quantifies the perceptual diversity of generated images by calculating the mean of MS-SSIM scores for pairs of these images [28]. This measurement allowed us to evaluate the level of variation and dissimilarity among the generated samples, providing insights into the model’s ability to produce diverse and distinct images.

### 2.5. 3D CNN PDAC Classifier

One of our objectives in creating synthetic data was to improve the performance of PDAC tumor identification. Currently, the limited available data are an obstacle. To test our developed 3D GAN model, we built and trained a 3D CNN classifier using the synthetic data generated by our 3DGAUnet model.

We developed a 17-layer 3D CNN model [29] to test if a 3D volumetric input was healthy pancreas tissue or had a tumor. The 3D CNN classifier has four 3D convoluted blocks (Conv 3D), with the first block consisting of 64 filters followed by 128, 256, and 512 filters, all with a kernel size of 3×3×3. Each Conv3D layer is followed by a max-pooling (MaxPool) layer with a stride of 2, ReLU activation, and batch normalization layer (Batch Norm). This 3D CNN model has four Con3D-MaxPool-BatchNorm blocks and is intended to capture visual features from coarse to fine. The final output first flattens the output of the last convolutional block and passes it to a fully dense layer with 512 neurons. A dropout layer with a tunable dropout rate follows, to prevent overfitting. The output is then passed to a 2-neuron dense layer with a sigmoid function for binary classification output. Because the input dimensions are 64×64×64, a relatively simple task, the architecture of the classifier was designed in a simple way to avoid the overparameterization problem, with 1,351,873 learnable parameters.

The binary classification performance was calculated from the confusion matrix. Given that TP, TN, FP, and FN correspond to true positive, true negative, false positive, and false negative, respectively, the results were measured using precision TP/(TP+FP), recall TP/(TP+FN), true positive rates TPR=TP/(TP+FN), and the false positive rates FPR=FP/(FP+TN). The area under the curve (AUC) was calculated from the receiver operating characteristic (ROC) curve, which was plotted as true positive rates against the false positive rates under different cutoffs or as the precision against recall.

## 3. Results

### 3.1. 3D Volumetric Tissue Data Generation

We trained our 3DGAUnet model separately using PDAC tumor and healthy pancreas data. These are referred to as the tumor and pancreas models. The tumor model was trained using PDAC tumor data, including 174 volumetric tumor data in Nifty format. The pancreas model was trained using healthy tissue data, including 200 volumetric data in Nifty format. Both input datasets resulted from the preprocessing steps outlined in Section 2.1.

Image augmentation, including image flipping and rotation, was performed on the training data. The augmented data for each volume were generated by rotating each volume on three axes in 12°, 24°, 36°, 48°, and 72° increments. All images for the tumor model were resampled to 1 mm isotropic resolution and trimmed to 32×32×32 size.All images for the pancreas model were resampled to 1 mm isotropic resolution and trimmed to 64×64×64 size, with pancreas tissue filling the entire cube.

The training procedure of any GAN model is inherently unstable because of the dynamic of optimizing two competing losses. For each model in this study, the training process saved the model weights every 20 epochs, and the entire model was trained for 2000 epochs. The best training duration before the model collapsed was decided by inspecting the generator loss curve and finding the epoch before the loss drastically increased. We trained our models with an NVIDIA RTX 3090 GPU. The optimal parameter set was searched within a parameter space consisting of batch size and learning rate, where the possible batch sizes included 4, 8, 16, and 32, and the possible learning rates included 0.1, 0.01, 0.001, 0.0001, and 0.00001.

**Figure 2:**
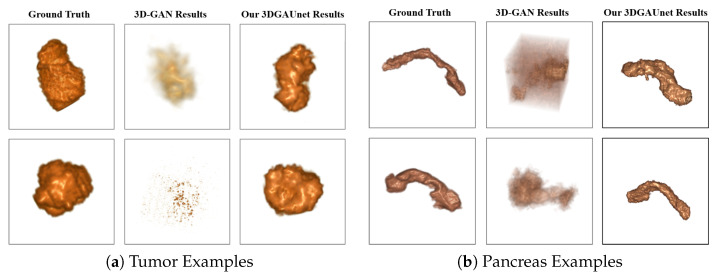
Examples of 3D volumes obtained from the different methods. Examples of 3D volume data of tumor (**a**) and pancreas (**b**) from the different methods. In each set of examples, the left, middle, and right columns correspond to ground truth data, synthetic data generated by 3D-GAN, and synthetic data generated by our 3DGAUnet, respectively. All 3D volumes are shown in volume rendering.

**Figure 3:**
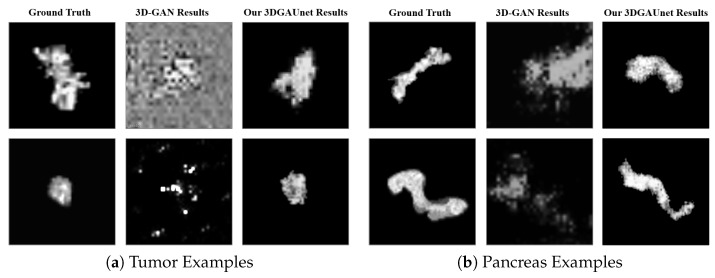
Examples of 2D slices in 3D volumes obtained from different methods. Examples of 2D in 3D volumes of tumor (**a**) and pancreas (**b**) from the different resources. In each set of examples, the left, middle, and right columns correspond to ground truth data, synthetic data generated by 3D-GAN, and synthetic data generated by our 3DGAUnet, respectively.

A set of 500 synthetic volumetric data were generated by the tumor and pancreas models separately. We first conducted a qualitative comparison between the training image sets and the synthetic image sets. We used volume rendering to visualize these datasets, to inspect the 3D results. Figure 2a shows examples of ground truth tumor volumes, synthetic tumors generated by the existing technique 3D-GAN [18], and synthetic tumors generated by our 3DGAUnet. We can see that when we trained our 3DGAUnet based on the group truth inputs, our model could generate synthetic tumors carrying realistic an anatomical structure and texture and capturing overall shape and details. Nonetheless, 3D-GAN either produced unsuccessful data or failed to generate meaningful results in capturing the tumor’s geometry. Figure 2b shows examples of the group truth pancreas volumes and synthetic pancreas volumes generated by 3D-GAN and our 3DGAUnet. By comparing the generated pancreas volumes with real medical images, we can see that our 3DGUnet could effectively synthesize a 3D pancreas to resemble actual anatomical structures. However, it was hard for 3D-GAN to generate anatomically plausible results, and a certain ambient noise was perceived in the generated volumes. We further examined the interior structures of the volumes generated by our 3DGUnet. Figure 3 shows the 2D slices of the ground truth, 3D-GAN, and our 3DGAUnet images from both tumor and pancreas models. The synthetic data produced by our 3DGAUnet model exhibited a high degree of fidelity to the ground truth, in terms of both internal anatomical structure and texture, compared to 3D-GAN. In certain instances, 3D-GAN failed to produce meaningful outcomes.

However, we can also observe marginal defects among pancreas generation, with tiny tissues surrounding the main tissue generated in the center. These defects were likely due to the irregular shape, different sizes, and direction of the pancreas, as well as the gradient learned from the input images batches at certain locations turning into noise.

In addition, we conducted a quantitative assessment of the outcomes produced by 3D-GAN and our 3DGAUnet models. A sample of 100 ground truth volumes and 100 synthetic volumes was selected randomly. In order to assess the quality of synthetic volumes, certain 2D image metrics, such as slice-wise FID and slice-wise PSNR, were computed. Besides the 2D metrics, 3D metrics such as batch-wise F3D, MMD^2^, and SSIM were calculated on the randomly selected volumetric data. Table 1 shows the values of 2D image metrics, slice-wise FID, and slice-wise PSNR on the sagittal (Sag), axial (Ax), and coronal (Cor) planes, to estimate the quality of the synthetic volumes, where scores were calculated using the center slice from 100 synthesized volumes and 100 ground truth volumes from the tumor model and pancreas model separately. Table 2 shows the values of the 3D volume metrics, batch-wise F3D, the MMD^2^, and the MS-SSIM. From the results, we can observe that our 3DGAUNet outperformed 3D-GAN in all metrics, suggesting that 3DGAUNet excelled at capturing the 3D shape and texture characteristics for both tumor and pancreas compared to 3D-GAN. In addition, it is evident that all the quantitative metrics in the pancreas model were better than the tumor model, especially on FID and F3D. This was probably caused by the difference in the training tasks, where the tumor model needed to learn both the texture and shape of the tumor, but the pancreas model was trained with pancreas-filled cubes to mostly learn the texture of the pancreas image.

### 3.2. 3D Volumetric Data Blending

From the synthesized data, we selected 100 pairs of synthetic tumors and synthetic pancreas tissue volumes. The paired data were used to evaluate three previously introduced blending methods. Quantitative evaluation was conducted by comparing the FID Score, while qualitative evaluation was conducted by visualizing the 2D slices. A total of 100 random abdominal CT images were cropped into 64 × 64 × 64 cubes as a negative sample set and then used to compare the 3D metrics with the blended volumes.

**Figure 4:**
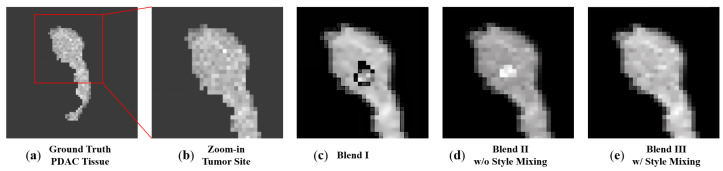
Comparison of the blending methods. For a ground truth PDAC tissue data (**a**), the image (**b**) provides a close-up view of the texture at the tumor site within the red box in (**a**), serving as a visual reference. The images (**c**–**e**) show the blend of a tumor into the healthy pancreas tissue using different blending methods. We can observe that Blend III had the best visual similarity.

Figure 4 shows the 2D slices of the blended images using the three different blending methods. It is evident that the direct copy-and-paste approach consistently yielded the least favorable results. The reason for this was that the tumor object was extracted with a simple threshold of the pixel value, and therefore the boundary of the tumor tissue may not have been as precise as needed. One can spot black pixels randomly appearing around the boundary of the tumor tissue and pancreas tissue. Whereas Blend III was visually closer to the ground truth tumor site and, on average, had a lower slice-wise FID score. Therefore, we employed Blend III as the blending method for the developed 3D GAN model. Table 3 compares the slice-wise FID values among the three blending methods and clearly shows that Blend III achieved the best slice-wise FID values.

### 3.3. Enhanced Training Dataset with Synthesized Data to Improve 3D PDAC Tumor Classification

We trained a 3D CNN classifier using two different dataset configurations, with or without adding the synthetic data, and compared the performance of the binary classification of PDAC between them. Adding synthesized data to the training data enlarged the training data and reduced the imbalance between positive and negative categories because, in practice, it is usually more difficult to access PDAC patient images than healthy pancreas images. We had a total of 174 PDAC tumor images and 254 healthy pancreas tissue images (a combination of 80 TCIA pancreas CT data and a non-tumorous portion of 174 PDAC data), all from real-world CT scans. All the input images were 1 mm isotropic resolution CT volume and trimmed to a size of 64×64×64. Out of all the data, 35 tumorous pancreas images and 51 healthy pancreas images, i.e., 20% of all data, were saved as the test dataset. We had two configurations for the training dataset. The first configuration (namely Config I) only contained real data for training, i.e., 139 tumorous pancreas images and 203 healthy pancreas images. The second configuration (namely Config II) included both the training set from Config I and synthesized data, i.e., 114 synthetic PDAC images and 50 synthetic healthy pancreas images. Both training datasets had the same test dataset for comparison. Config I was a baseline for real-world imbalanced data having a smaller size, and Config II used synthetic data to balance the entire dataset. Table 4 summarizes the training used in the two different configurations. For data augmentation, each CT scan underwent a random rotation along a single axis. The rotation angle was randomly selected from a set of options: 5°, 10°, 20°, and 40°. The direction of rotation, either clockwise or counterclockwise, was also randomly determined. The best parameters of each model were found with a grid search of a parameter space consisting of batch size and learning rate, where the batch sizes included 8, 12, and 16, and the learning rates included 0.001, 0.0001, and 0.00001. All the models were trained with an NVIDIA RTX 3090 GPU and validated using three-fold cross-validation.

**Figure 5:**
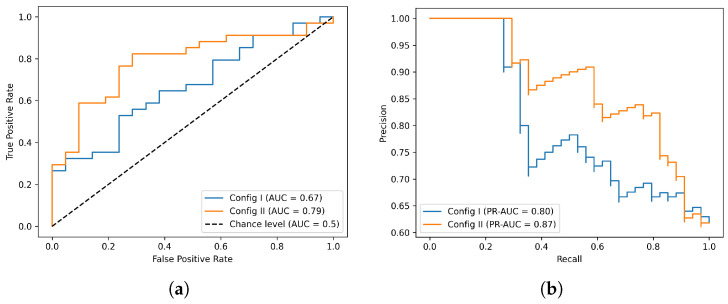
3D CNN classifier performance. (**a**) ROC curves and (**b**) PR curves with two configurations of training datasets.

Figure 5 shows the receiver operating characteristic (ROC) and precision-recall (PR) curves for the classification models. Config I had an ROC AUC (area under the curve) value of 0.67 and a PR AUC value of 0.80, while Config II had an ROC AUC value of 0.79 and a PR AUC value of 0.87. Given that the model was trained on a limited dataset, we assessed the classifier’s accuracy using three-fold cross-validation. In Configuration I, the average accuracy was 0.57 with a standard deviation of 0.07, while in Configuration II, the average accuracy was 0.67 with a standard deviation of 0.13. The analysis of the results indicated that, as the training dataset was enlarged and the training data in the two classes became more balanced, there was an observable increase in the AUC and precision–recall metrics. This finding implies that including synthesized data to solve issues related to training data quantity and class imbalances had a beneficial effect on the performance of the PDAC classifier. It is worth acknowledging that, despite advancements in utilizing large quantities and balanced training data, there is considerable room for enhancing the classifier’s overall performance. This might be achieved using a purpose-built 3D CNN model or by further refining the training methodology. By leveraging synthesized data, conducting extra research and analysis of the identification of supplementary components could improve the performance of classifiers and yield superior outcomes.

## 4. Discussion

In this work, we developed a 3D GAN model, 3DGAUnet, for tumor CT image synthesis; compared different blending methods for CT image synthesis; and explored the impact of our synthesis method on a real-world 3D CNN classifier for tumor diagnosis. 3DGAUnet was specifically designed for synthesizing clinical CT images by combining the 3D U-Net architecture with GAN principles, to generate realistic 3D CT scans of clinical data. To ensure its accuracy, we trained the model using 3D image data of both tumor tissue and healthy pancreas tissue. The quality of the synthesized images was rigorously evaluated using both qualitative and quantitative methods. The generated images demonstrated a more realistic texture than general the 3D-GAN with a CNN-based generator and exhibited the advantage of preserving spatial coherence better than 2D methods. One notable feature of our 3DGAUnet model was its ability to learn the inter-slice gradient, contributing to the overall realism of the generated data. The model also showcased consistent 2D FID values across all three axes, further affirming its capability to produce high-quality 3D images.

3DGAUnet uses preprocessed, fixed-size image cubes. Preprocessing still requires a significant amount of human labor and judgment, such as eliminating defects caused by high-density material markers and creating standardized volumes for each training dataset. All training datasets must also be manually annotated by medical professionals. More automatic methods would be desirable, to reduce the large cost of acquiring data for model training.

The blending method used in this work can insert the tumor image into the background tissue image at a fixed location. For the use case involving mesenchymal organs, the various locations of the tumor have distinct anatomical meanings, and the model must also acquire this information. One possible extension would be to include a segmentation module that can extract the features of each tissue type or organ from the original CT scans. By adopting this approach, the necessity of taking the blending technique into account might be eliminated, hence potentially mitigating the occurrence of faults.

The F3D score, which we implemented in this work for evaluation, is a naive extension of the original FID metric, and the activation vectors were extracted from an untrained, cold-started 3D CNN model. The stability of the F3D score in different image domains remains untested. In the future, it will be necessary to have an implementation of a benchmark dataset and rigorous testing procedures to establish a standardized measure.

The 3D CNN classifier simplifies the clinical diagnosis issue into a binary classification challenge, because only healthy normal pancreas images and pancreas images contain a tumor contrast. Multiple conditions may co-exist with the pancreas, such as non-cancerous lesions, inflammatory conditions or metastatic lesions, and vascular abnormalities. These obstacles remain unconsidered and will increase the cost of building such a model, due to a lack of high-quality, well-annotated data.

## 5. Conclusions

The 3DGAUnet model represents a significant advancement in synthesizing clinical tumor CT images, providing realistic and spatially coherent 3D data, and it holds great potential for improving medical image analysis and diagnosis. In the future, we will continue to address problems associated with the topic of computer vision and cancer. We would like to investigate reasonable usages of synthetic data and evaluations of data quality and usability in practice; for example, their effectiveness in training reliable classification models. We also plan to conduct reader studies involving domain experts (e.g., radiologists), to assess whether synthetic CTs can enhance diagnostic accuracy. This would offer additional clinical validation regarding the resemblance of synthesized CTs to real-world PDAC tumor characteristics. 

## Figures and Tables

**Table 1:** Performance based on 2D image quality metrics.

Tissue	Model	FID-Sag	FID-Ax	FID-Cor	PSNR-Sag	PSNR-Ax	PSNR-Cor
Tumor	3D-GAN	249.32	262.18	244.27	20.10	18.63	19.49
	3DGAUNet	198.23	202.44	188.66	16.52	17.76	17.16
Pancreas	3D-GAN	293.62	342.60	335.20	18.20	16.31	14.05
	3DGAUNet	287.75	435.72	327.41	12.73	7.21	9.42

**Table 2:** Performance based on 3D image quality metrics.

Tissue	Model	F3D	MMD^2^	MS-SSIM
Tumor	3DGAN	472.64	5571.90	0.86
	3DGAUNet	271.31	5327.32	0.81
Pancreas	3DGAN	889.40	8924.39	0.83
	3DGAUNet	872.33	9122.40	0.77

**Table 3:** Slice-wise FID values of blending methods.

Blending Methods	FID-Sag	FID-Ax	FID-Cor
Blend I	42.10	40.26	32.94
Blend II	21.01	35.82	12.62
Blend III	13.21	13.88	10.06

**Table 4:** Dataset configurations for classifier experiments.

	Training Set
Config I	139 True PDAC
	203 True Healthy Pancreas
Config II	139 True + 114 synthesized PDAC
	203 True + 50 synthesized Healthy Pancreas

## Data Availability

Data are contained within the article.

## References

[1] American Cancer Society (2022). Cancer Facts & Figures 2022.

[2] Program S.R. (2022). Surveillance, Epidemiology, and End Results (SEER) Program. SEER*Stat Database: Populations-Total U.S. (1969–2020) [Katrina/Rita Adjustment]-Linked To County Attributes-Total U.S., 1969–2020 Counties.

[3] Wu J., Qian T. (2019). A survey of pulmonary nodule detection, segmentation and classification in computed tomography with deep learning techniques. J. Med. Artif. Intell..

[4] Tandel G.S., Biswas M., Kakde O.G., Tiwari A., Suri H.S., Turk M., Laird J.R., Asare C.K., Ankrah A.A., Khanna N. (2019). A review on a deep learning perspective in brain cancer classification. Cancers.

[5] Sharif M.I., Li J.P., Naz J., Rashid I. (2020). A comprehensive review on multi-organs tumor detection based on machine learning. Pattern Recognit. Lett..

[6] Radiya K., Joakimsen H.L., Mikalsen K.Ø., Aahlin E.K., Lindsetmo R.O., Mortensen K.E. (2023). Performance and clinical applicability of machine learning in liver computed tomography imaging: A systematic review. Eur. Radiol..

[7] Xue Y., Tong W., Neri F., Zhang Y. (2022). PEGANs: Phased Evolutionary Generative Adversarial Networks with Self-Attention Module. Mathematics.

[8] Baltruschat I.M., Nickisch H., Grass M., Knopp T., Saalbach A. (2019). Comparison of deep learning approaches for multi-label chest X-ray classification. Sci. Rep..

[9] Coudray N., Ocampo P.S., Sakellaropoulos T., Narula N., Snuderl M., Fenyö D., Moreira A.L., Razavian N., Tsirigos A. (2018). Classification and mutation prediction from non–small cell lung cancer histopathology images using deep learning. Nat. Med..

[10] Li Y., Zhang H., Xue X., Jiang Y., Shen Q. (2018). Deep learning for remote sensing image classification: A survey. Wiley Interdiscip. Rev. Data Min. Knowl. Discov..

[11] Razzak M.I., Naz S., Zaib A. (2018). Deep learning for medical image processing: Overview, challenges and the future. Classification in BioApps: Automation of Decision Making.

[12] Li S., Song W., Fang L., Chen Y., Ghamisi P., Benediktsson J.A. (2019). Deep learning for hyperspectral image classification: An overview. IEEE Trans. Geosci. Remote Sens..

[13] Chu L.C., Park S., Kawamoto S., Yuille A.L., Hruban R.H., Fishman E.K. (2021). Pancreatic cancer imaging: A new look at an old problem. Curr. Probl. Diagn. Radiol..

[14] Si K., Xue Y., Yu X., Zhu X., Li Q., Gong W., Liang T., Duan S. (2021). Fully end-to-end deep-learning-based diagnosis of pancreatic tumors. Theranostics.

[15] Foret P., Kleiner A., Mobahi H., Neyshabur B. (2020). Sharpness-aware minimization for efficiently improving generalization. arXiv.

[16] Wei Z., Chen Y., Guan Q., Hu H., Zhou Q., Li Z., Xu X., Frangi A., Chen F. (2022). Pancreatic Image Augmentation Based on Local Region Texture Synthesis for Tumor Segmentation. Proceedings of the 31st International Conference on Artificial Neural Networks.

[17] Guan Q., Chen Y., Wei Z., Heidari A.A., Hu H., Yang X.H., Zheng J., Zhou Q., Chen H., Chen F. (2022). Medical image augmentation for lesion detection using a texture-constrained multichannel progressive GAN. Comput. Biol. Med..

[18] Wu J., Zhang C., Xue T., Freeman B., Tenenbaum J. Learning a probabilistic latent space of object shapes via 3d generative-adversarial modeling. Proceedings of the Advances in Neural Information Processing Systems.

[19] Antonelli M., Reinke A., Bakas S., Farahani K., Kopp-Schneider A., Landman B.A., Litjens G., Menze B., Ronneberger O., Summers R.M. (2022). The medical segmentation decathlon. Nat. Commun..

[20] Roth H., Farag A., Turkbey E.B., Lu L., Liu J., Summers R.M. (2016). Data From Pancreas-CT (Version 2). The Cancer Imaging Archive.

[21] Hounsfield G.N. (1980). Computed medical imaging. Science.

[22] Oktay O., Schlemper J., Folgoc L.L., Lee M., Heinrich M., Misawa K., Mori K., McDonagh S., Hammerla N.Y., Kainz B. (2018). Attention u-net: Learning where to look for the pancreas. arXiv.

[23] Zhang L., Wen T., Shi J. Deep Image Blending. Proceedings of the IEEE Winter Conference on Applications of Computer Vision.

[24] Kikinis R., Pieper S.D., Vosburgh K.G. (2013). 3D Slicer: A platform for subject-specific image analysis, visualization, and clinical support. Intraoperative Imaging and Image-Guided Therapy.

[25] Heusel M., Ramsauer H., Unterthiner T., Nessler B., Hochreiter S. Gans trained by a two time-scale update rule converge to a local nash equilibrium. Proceedings of the Advances in Neural Information Processing Systems.

[26] Szegedy C., Vanhoucke V., Ioffe S., Shlens J., Wojna Z. Rethinking the inception architecture for computer vision. Proceedings of the IEEE Conference on Computer Vision and Pattern Recognition.

[27] Gretton A., Borgwardt K.M., Rasch M.J., Schölkopf B., Smola A. (2012). A kernel two-sample test. J. Mach. Learn. Res..

[28] Odena A., Olah C., Shlens J. Conditional image synthesis with auxiliary classifier gans. Proceedings of the International Conference on Machine Learning.

[29] Tran D., Bourdev L., Fergus R., Torresani L., Paluri M. Learning spatiotemporal features with 3d convolutional networks. Proceedings of the IEEE International Conference on Computer Vision.

